# SpheronizaTor: Spherical Voxelization for Interpretable Protein Microenvironment Modeling

**DOI:** 10.34133/csbj.0076

**Published:** 2026-05-07

**Authors:** Jose Cleydson Ferreira Silva, Matthew Richardson, José D. D. Cediel-Becerra, Layla Schuster, Raquel Dias

**Affiliations:** ^1^ Department of Microbiology and Cell Science, Institute of Food and Agricultural Sciences, University of Florida, Gainesville, FL, USA.; ^2^ Department of Chemical Engineering, Herbert Wertheim College of Engineering, University of Florida, Gainesville, FL, USA.

## Abstract

Artificial intelligence (AI) has expanded the reach of structural biology by enabling models to extract biochemical and geometric features directly from 3-dimensional (3D) protein structures. Yet, the effectiveness of these models depends critically on how protein environments are encoded. Most existing volumetric representations rely on Cartesian voxel grids derived from smoothed atomic densities, an approach that offers broad applicability but struggles to reconcile rotational invariance, residue-level specificity, and explicit biochemical detail. We present SpheronizaTor, a residue-centered voxelization framework for protein structures that builds local spherical voxel maps centered on each residue. Each spherical map encodes atom types, covalent bonding information, and whether atoms belong to the central residue or neighboring residues. By producing one voxel representation per residue, SpheronizaTor emphasizes the structural and functional granularity through which proteins organize catalysis, recognition, and stability. The combination of spherical alignment with chemically explicit feature channels enables richer interpretability and enhances compatibility with 3D convolutional and hybrid neural architectures. Designed specifically for proteins and engineered for extensibility, SpheronizaTor provides a voxelization strategy that is both chemically realistic and computationally efficient. The residue-centric approach bridges the gap between global volumetric encoders and graph-based models, offering a versatile foundation for downstream tasks such as mutation effect prediction, binding site analysis, and structural comparison across protein families.

## Introduction

Deep learning has profoundly transformed structural bioinformatics, revealing complex geometric and physicochemical patterns in macromolecular data through advances in geometric deep learning, graph neural networks, and large-scale protein modeling frameworks [1–3]. Breakthrough methods such as AlphaFold and RoseTTAFold have showcased the power of data-driven approaches to predict protein structures with remarkable accuracy and at scale, fundamentally reshaping our understanding of protein folding and function [4,5]. Beyond structure prediction, machine learning techniques are now widely employed to dissect local protein environments, pinpoint functional sites, and model molecular interactions with striking resolution [6]. Yet, despite this rapid progress, the representation of protein microenvironments remains a critical bottleneck, as conventional encoding strategies often struggle to capture the intrinsic geometric invariances and local structural heterogeneity required for robust learning. This challenge motivates the development of new representation frameworks that more faithfully encode residue-level context while preserving structural interpretability. Different methods have been developed to encode proteins in ways that balance biological interpretability, rotational invariance, computational efficiency, and rich biochemical detail [7,8]. Such approaches include global Euclidean grid-based representations and Cartesian voxel grids based on atomic densities. For example, volumetric frameworks such as PyUUL [9] convert macromolecular structures into 3-dimensional (3D) grids through Cartesian voxelization and continuous atomic density functions. Similarly, HTMD [10] volumetric featurizer constructs Gaussian smoothed atom density grids for proteins and ligands, producing standardized volumetric tensors for machine learning pipelines. Likewise, libmolgrid [11] generates voxelized atom-type density fields directly from molecular files and serves as the primary volumetric engine for learning-based docking frameworks. Within classical docking pipelines, AutoGrid, part of the AutoDock4 [12] suite, creates atom-type specific 3D interaction maps that operate as early voxel representations of macromolecular environments.

However, these and other tools still present important challenges: (a) global Euclidean grid-based representations suffer from computational efficiency and often smooth away atom-level detail [2]; (b) existing voxel encodings are orientation-sensitive, often requiring additional orientation normalization modules [13]; (c) density-based encodings primarily capture continuous atomic occupancy, providing limited access to discrete biochemical attributes such as residue membership (e.g., central residue from its surroundings) or bonding information, and (d) do not separate residue-level context. These limitations point to the need for an alternative representation that aligns with the biochemical architecture of proteins, where the fundamental units of information are residue-level features underlying tasks such as binding site recognition, protein–ligand interaction prediction, mutational effect modeling, and microenvironment comparison. A framework that explicitly centers the coordinate system on residues provides a more natural way to capture local context, reduces the rotational variability inherent to Cartesian grids, and mirrors the hierarchical organization through which proteins mediate recognition and catalysis.

To address these limitations, we introduce SpheronizaTor, a residue-centered voxelization framework that constructs local voxel representations within residue-aligned coordinate frames. The method integrates geometric normalization, explicit separation between the central residue and its surrounding environment, and multi-channel encoding of atom types and bond features. This representation is made consistent by normalizing residue-centered coordinates. It reduces orientation dependence without imposing full rotational invariance. Consequently, it remains aligned with the local geometry of protein interactions [14]. Within each spherical grid, SpheronizaTor encodes a multi-channel feature space capturing fundamental atom types of hydrogen (H), carbon (C), nitrogen (N), oxygen (O), phosphorus (P), and sulfur (S), bonding information, and annotations distinguishing atoms from the central residue versus atoms from neighboring residues. By integrating both chemical composition and structural connectivity, it enables models to learn richer, more interpretable representations of local protein environments. Benchmarking results demonstrate that SpheronizaTor-derived representations consistently outperform conventional voxelization approaches. These results highlight the importance of residue aligned encoding. This strategy enables more accurate capture of biologically meaningful protein microenvironments. The framework extends existing volumetric encoders and provides a versatile, extensible foundation for a wide range of downstream machine learning [15] tasks in structural bioinformatics. SpheronizaTor is an open-source Python software and can be installed via Conda or pip from its GitHub repository (https://github.com/Dias-Lab/spheronizator)

## Results and Discussion

### Residue-centric spherical voxelization of protein microenvironments

**Fig. 1.**
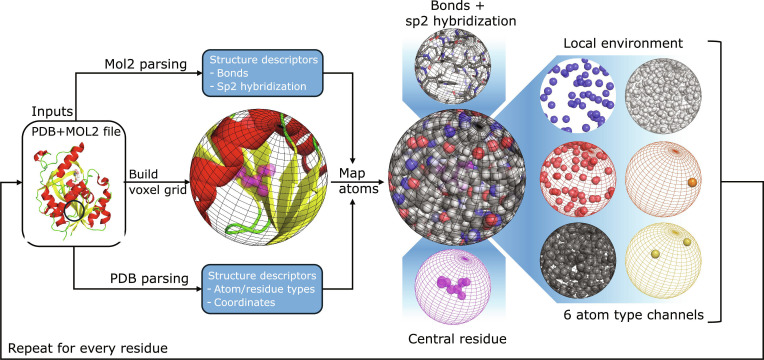
SpheronizaTor workflow for residue-centered spherical voxelization. Protein structures are parsed from paired PDB and MOL2 files to obtain Cartesian coordinates, atom types, and covalent bond information. For each residue, SpheronizaTor constructs a local spherical voxel grid and maps all surrounding atoms into this coordinate frame. MOL2-derived biochemical descriptors, including bond order and hybridization, are integrated with PDB-derived spatial and residue annotations. Each spherical environment is decomposed into multi-channel voxel representations consisting of 6 atom-type channels (H, C, N, O, P, S) and additional bonding descriptors.

SpheronizaTor introduces a residue-centric voxelization strategy that captures local biochemical environments with high resolution and flexibility. Unlike continuous density-based encoders (e.g., PyUUL, HTMD, and libmolgrid), SpheronizaTor constructs spherical voxel grids centered on each residue, encoding atom-type channels (H, C, N, O, P, S), covalent bonds, and flags distinguishing atoms belonging to the central residue from neighboring residues (Fig. 1). This integrated representation of chemical composition and structural connectivity provides a rich, residue-specific feature space for downstream modeling.

**Fig. 2.**
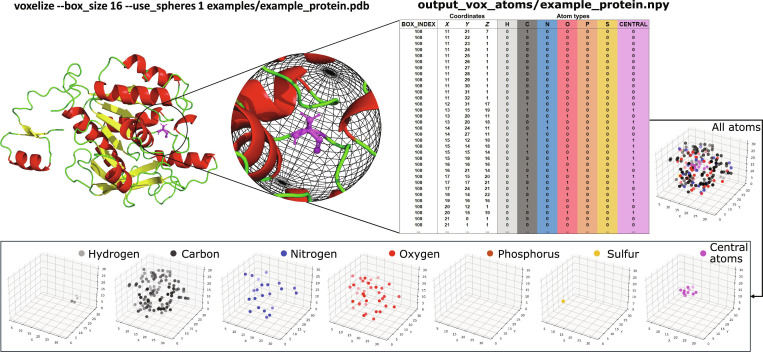
Example outputs for residue-entered spherical voxelization of MetA from *E. coli* (PDB ID 7CBE). For each residue-centered sphere, SpheronizaTor produces a multidimensional NumPy array (output_vox_atoms/example_protein.npy) that stores the 3D voxel coordinates together with binary occupancy channels for the 6 canonical atom types (H, C, N, O, P, S) and a flag indicating whether each atom belongs to the central residue or to neighboring residues. The table and 3D scatterplots illustrate the voxelized microenvironment for sphere 108, corresponding to central residue 108 (alanine) of MetA, with separate panels highlighting individual atom-type channels as well as all atoms combined.

**Fig. 3.**
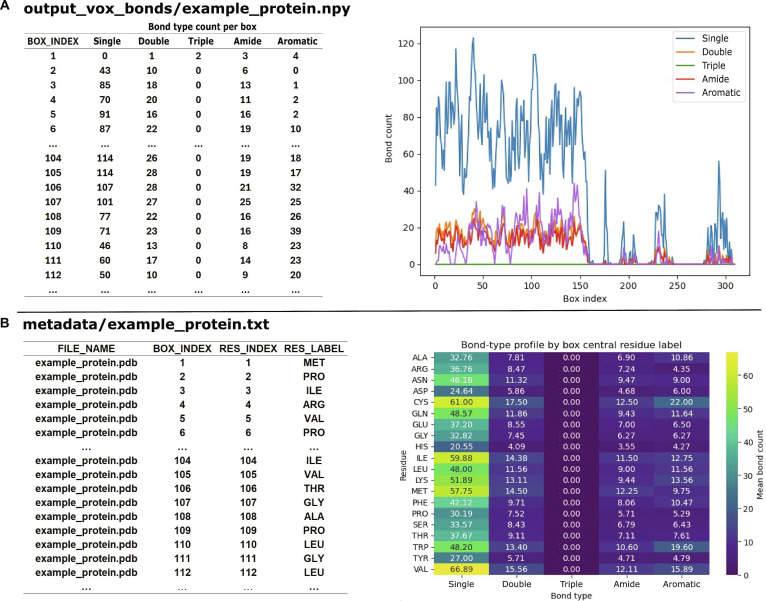
Example outputs for bond type counts and metadata outputs generated for MetA from *E. coli* (PDB ID 7CBE). (A) For each residue-centered sphere, SpheronizaTor summarizes MOL2-derived covalent connectivity into bond type counts (single, double, triple, aromatic, amide) and stores these as a per box bond feature matrix (e.g., output_vox_bonds/example_protein.npy), which can be visualized as bond count profiles across box indices. (B) The accompanying metadata file records, for each box, the protein identifier, box index, residue index, and residue label (e.g., metadata/example_protein.txt).

As an example, we visualized the residue-centered voxelization of MetA, a temperature-sensitive enzyme initiating the methionine biosynthesis pathway and often used as a model system for studying thermostable protein behavior. We selected MetA for its scientific relevance, moderate size, and well-characterized structure, which makes it suitable for testing residue-level representations. For central residue 108 (alanine), SpheronizaTor maps the surrounding microenvironment onto a spherical grid and outputs a structured feature array containing voxel coordinates, atom-type occupancies, and central residue annotations (Fig. 2). The corresponding bond feature matrix summarizes local covalent connectivity, enabling comparison of bonding patterns across residues and facilitating residue-level statistical analysis (Fig. 3).

### Benchmark results and practical advantages

**Fig. 4.**
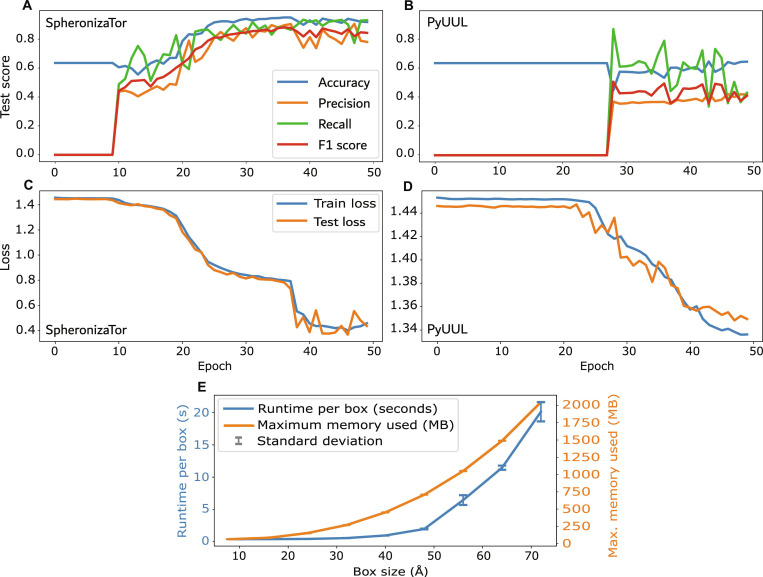
SpheronizaTor outperforms PyUUL in α-helix classification and shows efficient scaling. (A and B) Test set performance metrics over 50 epochs using fully convolutional 3D CNN for α-helix classification, showing SpheronizaTor (A) versus PyUUL (B). Lines: accuracy (blue), precision (orange), recall (green), F1 score (red). Same model architecture and training procedure was applied to SpheronizaTor and PyUUL representations for fair feature comparison. (C and D) Training (blue) and test (orange) loss curves over 50 epochs for SpheronizaTor (C) and PyUUL (D). (E) SpheronizaTor computational performance. We benchmarked runtime and memory usage across multiple box sizes in a single CPU core with 8 GB available RAM.

Models trained on SpheronizaTor features showed higher accuracy, F1 scores and Dice coefficients than those trained on PyUUL features (Table S1 and Fig. 4). This improvement may reflect SpheronizaTor’s residue-centered encoding, which can better preserve local geometric detail and chemical context, thereby improving performance. These results show that representation design matters as much as model architecture for protein microenvironment tasks.

When compared with density-based frameworks such as PyUUL [9] and other tools, which emphasize smooth volumetric fields derived from differentiable kernels, SpheronizaTor preserves discrete biochemical asymmetries that can be lost in continuous representations (Table 1). Density-based encodings excel at capturing global molecular shape but often blur atom identity and covalent topology. SpheronizaTor, by contrast, explicitly encodes these distinctions, allowing models to access fine-grained structural and chemical signals that are critical for tasks involving residue-level inference.

**Table 1.** Feature comparison of voxelization software

Feature/capability	SpheronizaTor	PyUUL	GNINA/MoleculeKit (continuous density encoders)	PyTorch geometric/DGL-LifeSci (graph-based models)
Residue-centered representation	X			
Atom-level representation	X	X	X	X
Explicit atom-type channels	X	X		X
Central vs. neighbor residue separation	X			
Spherical voxel grids	X			
Cartesian voxels	X	X	X	
Continuous density fields			X	
Bond feature encoding	X			X
MOL2 chemical typing	X			
Multi-channel voxel features	X	X		
Rotationally normalized frames	X			
Environment specificity per residue	X			
Global voxel representation		X	X	
Graph topology encoding				X
Demonstrated integration with 3D CNNs	X	X	X	
Requires PDB + MOL2	X			
Low computational cost	X		X	X

Another defining feature is the generation of one voxelized environment per residue rather than a single global grid. That enables machine learning models to learn localized biochemical signatures, interaction neighborhoods, and context-dependent structural patterns. This localized representation also has low computational cost, enabling voxelization of an entire protein (approximately 70-Å box size) in under 25 s on a single central processing unit (CPU) core while using only about 2 GB of RAM (Fig. 4). The integration of MOL2-derived bond information further strengthens the representation by unifying geometrics with covalent structure, avoiding the need for post hoc graph reconstruction.

SpheronizaTor is designed for seamless interoperability with Python-based deep learning frameworks such as PyTorch [16,17], JAX [17], TensorFlow [17,18], as well as traditional machine learning pipelines and 3D structure analysis libraries. To support large-scale inference and dataset creation, SpheronizaTor supports exporting NumPy arrays, enabling memory efficient storage of voxelized biochemical features for proteins ranging from small domains to multi-chain complexes. From a computational perspective, SpheronizaTor maximizes efficiency by computing voxel grids once per protein and reusing them across all residues, reducing redundant memory allocations (Fig. 4).

Together, these features position SpheronizaTor as a powerful and extensible framework for residue-level protein representation learning. By combining spherical coordinate alignment with a comprehensive and customizable feature set, it provides interpretable volumetric encodings that capture both chemical identity and interaction context [15]. The residue-focused voxelization paradigm is well suited for applications requiring detailed structural modeling, such as mutation modeling, binding site characterization, and protein ligand interaction prediction, and integrates naturally into modern deep learning workflows.

## Methods

### Use case dataset

The protein structures used in this study were obtained from the dataset distributed with the PyUUL framework, originally curated for voxel-based protein classification tasks [9]. All structures were downloaded from the publicly available repository at https://bitbucket.org/grogdrinker/pyuul, specifically from the classification_data directory (https://bitbucket.org/grogdrinker/pyuul/src/master/dataPaper/classification_data/). The dataset comprises experimentally resolved protein structures in PDB format that have been preprocessed to ensure consistency in atomic representation and compatibility with voxelization-based machine learning workflows.

### Protein structure acquisition and preprocessing

Protein structural data are obtained from paired Protein Data Bank (PDB) [19] to generate voxel-based biochemical encodings. Input PDB files are parsed using the Biopython [20] structure class, which extracts, for each atom, the Cartesian coordinates, chain identifier, residue name, and residue index. PDB files are converted to MOL2 format with matching atom indices and coordinates to extract additional chemical details. SpheronizaTor’s mol2parser module then parses additional information from the MOL2 file, including (a) interatomic bonds, (b) SYBYL atom types encoding atom’s hybridization state and chemical environment according to the Tripos force field (e.g., *C.3* denotes an sp^3^-hybridized tetrahedral carbon, *C.2* an sp^2^ trigonal planar carbon, *N.pl3* a planar sp^2^ amide nitrogen, and *O.co2* a carboxylate oxygen with resonance delocalization), and (c) simplified atom categories (*C.3* → C, *N.pl3* → N, *O.co2* → O). Structures with missing or inconsistent annotations are excluded. The resulting preprocessed structure consists of a chemically annotated, residue-aware atomic dataset that integrates spatial, biochemical, and topological information into a single, coherent representation. This representation is used to construct residue voxel grids in subsequent steps in SpheronizaTor, providing machine learning-ready features that can be directly leveraged by end users in downstream artificial intelligence (AI) and data-driven modeling pipelines. The resulting unified atomic dataset with spatial, chemical, and bond information is then used to build residue-centered spatial representations.

### Construction of residue-centered spatial representations

For each residue, the backbone atoms (N, Cα, C) define both position (centroid) and orientation (N→Cα→C vector aligned to canonical axes) using the get_boxProjection function. All atomic coordinates are transformed into this residue-centered frame. This coordinate normalization removes global positional dependence and allows residue-centered environments from different proteins, or from alternative conformations of the same protein, to be compared directly. A uniform voxel lattice [21] is then generated and centered on each residue using a box size *B* and voxel spacing *s*, producing a discrete 3D grid. Atoms are assigned to the nearest voxel, and atoms outside the bounding box are excluded. Atoms within the volume are labeled as either central-residue or neighboring-residue atoms. When the --use_spheres command line option is enabled, the buildSphere function applies a spherical neighborhood constraint where only atoms within a radial distance ‖x‖ ≤ B from the centroid are retained (see https://dias-lab.github.io/spheronizator/cli.html). This spherical masking defines the residue-centered local microenvironment, while the underlying voxel discretization remains Cartesian. The resulting spatial encoding serves as the basis for adding biochemical and bonding features.

### Assembly of biochemical voxel features and output format

After projection into residue-centered spatial frames, atoms are assigned to the nearest voxel and 6 occupancy channels are created for H, C, N, O, P, and S atom types. Each voxel also records whether atoms belong to the central residue or neighboring residues, producing another 6D tensor that separates intrinsic residue structure from environmental contributions. This dual-channel representation captures fine-grained biochemical asymmetry that is typically obscured in density-based voxel encoders. In parallel, a bond feature tensor is constructed to summarize local covalent topology from the MOL2 file including bond type counts (single, double, triple, amide, aromatic).

All residue-level voxel and bond features are organized into a unified data structure for use by machine learning models and structural analysis tools. For each protein, SpheronizaTor produces 2 main outputs: (a) an occupancy tensor with shape (number of residues × length × width × height × 6 × 2), which encodes voxel positions for each residue environment, 6 atom types (H, C, N, O, P, S), and central-versus-neighboring residue flag; (b) a bond tensor (number of residues × 5), containing counts of single, double, triple, aromatic, and amide bonds per residue. The second is a bond feature matrix, summarizing local covalent topology through counts and distributions of bond types. These outputs are stored in a dataWrapper object with metadata and coordinate references and can be exported as Numpy (.npy) files.

### α-Helix classification use case

Protein secondary structure annotations were obtained with define secondary structure of proteins (DSSP) through the MDAnalysis package using only protein atoms from 483 structures retrieved from PyUUL’s example dataset [22]. Residues classified as α-helices (label “H”) were assigned a label of 1, and all others a label of 0.

We developed a 3D convolutional neural network (3D CNN) in PyTorch to predict α-helix residues from SpheronizaTor representations as a use case demonstration [23,24]. The architecture processes multi-channel 3D inputs (*X*∈*R*^*C* × *D* × *H* × *W*^, where *C* is the number of channels and *D* × *H* × *W* defines the spatial grid) through a stem block of 3 × 3 × 3 convolution (padding = 1), group normalization, and LeakyReLU (α = 0.1), preserving resolution while increasing channel depth. Feature extraction uses stacked residual 3D blocks, each with two 3 × 3 × 3 convolutions, normalization, activation, and identity/projection skip connections. A context module applies dilated 3D convolution (dilation = 2), with outputs concatenated to encoder features. Fused features pass through a convolutional head, 1 × 1 × 1 convolution, global average pooling, 2 linear layers, and LeakyReLU activation.

The output layer produces logits that are converted to predicted probabilities via sigmoid and binarized at threshold 0.5 for evaluation. ε = 1e−8 was used for numerical stability. Training and evaluation used an 80%–20% train–test split (386 training structures and 97 test structures) and were carried out for 50 epochs. Training used the AdamW optimizer with learning rate 1 × 10^−3^ and weight decay 1 × 10^−4^, and optimization was guided by a combined binary cross-entropy (BCE) and Dice loss (BCE + Dice loss) between predicted probabilities and ground-truth labels [25]. Test set evaluation computed mean accuracy, precision, recall, F1 score, Dice coefficient, and intersection over union (IoU) [26] from sigmoid predictions threshold at 0.5. Same model architecture and training procedure was applied to SpheronizaTor and PyUUL representations for fair feature comparison.

### Runtime and memory benchmarks

The voxelize command within SpheronizaTor Python utility was executed for Homoserine O-succinyltransferase (MetA) from *Escherichia coli* (PDB ID 7CBE), using a box size of 16 Å (sphere radius), spherical mode enabled (--use_spheres 1), and a default voxel spacing of 1 Å. All executions were performed on a HiPerGator computational node at the University of Florida, equipped with an AMD EPYC 9655 P 96-core processor, 756 GB of RAM, and 3 NVIDIA L4 Tensor GPUs (23 GB VRAM each, Turing architecture, CUDA Compute). For benchmarking purposes, only a single CPU core and 8 GB of RAM were utilized, without GPU acceleration. The Spheronizator program was executed for the *MetA* protein, and runtime performance and memory usage were profiled using the Unix/Linux time command with the format specifier time -f “%e, %M”, which reports the elapsed runtime (in seconds) and peak memory consumption (in kilobytes), respectively. The mean runtime per sphere/box generation was computed from these measurements.

### Limitations and future directions

While SpheronizaTor provides an interpretable framework for residue-centered protein representation, several limitations remain. First, the present study focuses on voxel-based encoding and does not explicitly include physicochemical interaction features such as contact surfaces, orientation-specific interactions, or solvent accessibility, all of which have been shown to improve protein model quality assessment methods like DeepUMQA-PA and EquiRank [27,28]. These approaches underscore the value of incorporating geometric and physics-based descriptors, especially for modeling protein–protein interfaces and multi-chain assemblies [29]. Second, although the proposed representation is compatible with modern machine learning architectures, including 3D CNNs, it does not explicitly enforce rotational equivariance, as in recent E (3)-equivariant neural networks [30]. Future work could explore equivariant learning or hybrid representations that combine voxel- and graph-based features. Finally, evaluation here is restricted to a proof-of-concept downstream task. Future studies will test broader applications, including protein–protein interaction prediction, to adapt to molecular dynamic analysis [31], mutation impact modeling, and protein complex quality assessment, where residue-level microenvironment representations may provide additional predictive value [32].

## Data Availability

The SpheronizaTor software is freely available on GitHub at https://github.com/Dias-Lab/spheronizator and can also be installed via pip (https://pypi.org/project/spheronizator/). Detailed documentation is available at https://dias-lab.github.io/spheronizator/.
